# Assessment of Biomedical Waste Management Practices and Risk Perception Among Healthcare Workers in South India

**DOI:** 10.7759/cureus.83584

**Published:** 2025-05-06

**Authors:** Yogesh Manickam Dominic Savio, Devi Krishna Ravichandran, Hariharan Surathkumaar, Yashvanthan Vinjmur Ragavan, Priya Santharam, Ashni Bhandari, Krishna Prasanth

**Affiliations:** 1 Community Medicine, Sree Balaji Medical College and Hospital, Chennai, IND; 2 General Practice, Kauvery Hospital, Chennai, IND; 3 Pharmacology, Saveetha Medical College and Hospital, Chennai, IND; 4 Microbiology, Sree Balaji Medical College and Hospital, Chennai, IND; 5 Epidemiology and Public Health, Sree Balaji Medical College and Hospital, Chennai, IND

**Keywords:** biomedical waste, healthcare workers, hospital safety, india, infection control, needlestick injuries, occupational exposure, risk perception, tertiary care, waste segregation

## Abstract

Introduction

Inefficient biomedical waste (BMW) management in healthcare settings can expose patients, workers, and the environment to significant risks. This study aimed to assess BMW segregation practices, occupational risk perception, training exposure, and sharps injury incidence among healthcare workers (HCWs) across multiple tertiary care hospitals in South India.

Methods

A cross-sectional observational study was conducted over six months across four randomly selected tertiary care teaching hospitals in Chennai, Tamil Nadu. A total of 356 healthcare workers comprising 84 (23.6%) doctors, 186 (52.2%) nurses, and 86 (24.2%) housekeeping staff were selected using stratified random sampling. Data were collected via a pre-validated, structured questionnaire (55 items), on-site observations, and semi-structured interviews. Risk perception was measured using a five-point Likert scale, and a simplified infection risk model adapted from the UK Environmental Agency was applied. Data were analyzed using SPSS version 26.0 (IBM Corp., Armonk, NY, USA), employing chi-square tests and one-way ANOVA.

Results

Overall, 257 (72.1%) of participants demonstrated correct knowledge of BMW segregation protocols, with highest accuracy among nurses (142, 76.3%) and lowest among doctors (53, 63%). Field observations revealed inconsistent compliance, particularly in high-volume areas. Risk perception was highest for environmental contamination (mean score: 4.30) and waste worker exposure (4.08), but significantly lower for patients and visitors. A total of 41 sharps injuries were reported, with underreporting observed among doctors. Training participation varied significantly across roles: only 26 (31%) doctors were trained compared to 138 (74.2%) nurses and 76 (88.3%) housekeeping staff. Training was strongly associated with both risk perception and segregation accuracy (p < 0.05).

Conclusion

This multi-center study highlights critical gaps in BMW knowledge, risk perception, and training, particularly among doctors. Despite awareness, compliance remains inconsistent, underscoring the need for mandatory training, improved monitoring, and institution-wide reinforcement of biomedical waste protocols to reduce health and environmental hazards.

## Introduction

Biomedical waste (BMW) generated in healthcare settings presents a substantial risk to both human health and the environment when not managed appropriately. According to the World Health Organization (WHO), approximately 15% of all healthcare waste is classified as hazardous due to its infectious, toxic, or radioactive properties [1]. Inadequate handling and segregation of this waste can result in the transmission of bloodborne pathogens, including HIV, hepatitis B (HBV), and hepatitis C (HCV), as well as environmental contamination.

India, with its rapidly expanding healthcare infrastructure, produces significant volumes of biomedical waste. Despite the enactment of the Biomedical Waste Management Rules in 2016 and subsequent amendments, compliance and enforcement remain inconsistent across institutions [2,3]. Studies in both government and private hospitals in India have highlighted challenges such as poor segregation, inadequate training, and limited awareness among staff involved in waste handling [4].

The risk perception of healthcare workers (HCWs) plays a pivotal role in determining adherence to safe practices. Past research has shown that risk perception varies by occupational role, with housekeeping staff often underestimating the dangers associated with direct exposure to biomedical waste [5,6]. A study by Ferreira and Teixeira in Portugal [7] demonstrated that daily contact with healthcare waste strongly influences both segregation practices and perceived occupational risk.

Given these concerns, this study was designed to assess BMW management practices, risk perceptions, and occupational exposure risks among doctors, nurses, and housekeeping personnel across four tertiary care teaching hospitals in South India. These factors are essential for developing targeted interventions to improve compliance, reduce health hazards, and ensure safe waste handling in clinical settings.

## Materials and methods

Study design and duration

This was a hospital-based, cross-sectional observational study conducted over a duration of slightly more than six months, from 20/5/2024 to 13/12/2024, in Chennai, Tamil Nadu. The objective was to assess BMW segregation practices, occupational risk perception, training exposure, and sharps injury incidence among healthcare workers in tertiary care teaching hospitals.

Study setting and hospital selection

The study was conducted in four tertiary care teaching hospitals located in the metropolitan region of Chennai. These hospitals were selected based on predefined eligibility criteria, including a minimum bed capacity of 500, provision of multispecialty inpatient and outpatient services, and accreditation as teaching hospitals affiliated with a recognized medical college

A list of eligible hospitals was compiled, and simple random sampling without replacement was applied using a computer-generated random number sequence. This approach ensured equal selection probability for each institution and minimized selection bias. Inclusion of multiple hospitals was intended to improve the external validity of the study by capturing inter-institutional variability in BMW management practices.

Participant selection and stratified sampling

Following the selection of hospitals, HCWs were recruited using a proportionally stratified sampling technique. The study population comprised three predefined occupational strata of doctors (including interns, postgraduate residents, and consultants, nurses and housekeeping staff).

Each participating hospital provided anonymized lists of active staff members in the above categories. These served as the sampling frames for that hospital. Based on the relative size of each staff category, predefined proportional quotas were calculated to ensure real-world representation of the hospital workforce.

Participants within each stratum were selected using simple random sampling, employing a computer-generated random number table to maintain objectivity. Where official rosters were unavailable or dynamic due to shift schedules, on-site recruitment was carried out using proportional quota sampling until target numbers were reached for each category.

Inclusion and exclusion criteria

The criteria for inclusion and exclusion are given below (Table 1).

**Table 1:** Inclusion and Exclusion Criteria HCWs: Healthcare Workers

Inclusion Criteria	Exclusion Criteria
HCWs with at least six months of continuous service	Administrative or non-clinical staff not involved in waste handling
Direct or indirect involvement in biomedical waste handling	HCWs on long-term leave or unavailable during the data collection period
Provided informed written consent	

Sample size and distribution

A total of 356 healthcare workers were enrolled across the four hospitals, with 84 doctors representing 23.6% of the study, 186 nurses representing 52.2%, and 86 housekeeping staff representing 24.2% of the study population.

This sample size was calculated to achieve 95% confidence and a 5% margin of error, and it included approximately 30% of the total workforce involved in BMW management across all sites, based on feasibility and proportional representation within each staff category. A non-response rate was not factored into the initial sample size estimation; however, full participation was achieved from all 356 selected healthcare workers, and no data were excluded, thereby preserving the intended statistical power.

Data collection tools and procedures

Structured Questionnaire

A multi-method approach was adopted to ensure data triangulation. A pre-validated, self-administered questionnaire was distributed in English and structured into four domains comprising 55 items. These included knowledge of BMW segregation as per the Biomedical Waste Management Rules 2016, occupational risk perception assessed using a 5-point Likert scale, history of needlestick or sharps injuries, and participation in formal BMW training (Appendix 1). The questionnaire was delivered digitally using Google Forms, accessible via mobile or computer devices. Data was collected across departments and shifts to minimize sampling bias. Knowledge of BMW segregation was evaluated using a 20-item section, with each item representing a specific type of biomedical waste and its appropriate disposal route, as outlined in the Biomedical Waste Management Rules, 2016. Participants were asked to identify the correct color-coded bin for each item. A score of 15 or more correct answers out of 20 (≥75%) was used to classify respondents as having "correct knowledge."

On-Site Observations

Structured field observations were carried out using a standardized checklist derived from WHO and Biomedical Waste Management Rules 2016. Key parameters assessed included the availability and correct use of color-coded bins, use of personal protective equipment, on-site waste storage, and intra-facility transport procedures. Observations were conducted in clinical units, intensive care units, operating theatres, and laboratories.

Semi-Structured Interviews

To explore organizational challenges and compliance issues, semi-structured interviews were conducted with biomedical waste management officers, nursing supervisors, and housekeeping in charges. These interviews explored policy implementation, staff compliance, and logistical challenges at the institutional level.

Risk assessment modeling

To estimate the probability of infection following sharps injuries, a simplified risk model adapted from the UK Environmental Agency 2002 was used. While India does not publish a formal equivalent, this model is consistent with infection control guidelines from National AIDS Control Organization (NACO), Indian Council of Medical Research (ICMR), and Ministry of Health and Family Welfare (MoHFW). Variables were adjusted using national seroprevalence estimates for HIV, HBV, and HCV, observed sharps injury rates in the study population, and vaccination status and post-exposure prophylaxis (PEP) access of the exposed healthcare workers. This model provided a contextual infection risk estimate for different occupational groups.

Data analysis

Responses from Google Forms were exported and analyzed using IBM SPSS version 26.0 (IBM Corp., Armonk, NY, USA). Descriptive statistics were computed for demographic and practice variables. Inferential tests included chi square tests for categorical comparisons such as training versus role and one-way ANOVA for comparing mean risk perception scores across staff categories. A p value of less than 0.05 was considered statistically significant.

## Results

Participant characteristics

A total of 356 healthcare workers were included in the study, comprising 84 doctors (23.6%), 186 nurses (52.2%), and 86 housekeeping staff (24.2%).

The mean age of participants was 34.7 ± 7.6 years, with a female predominance among nurses of 153 (82.3%) and a male majority among doctors of 52 (61.9%). All participants had more than six months of employment, with 240 (67.4%) reporting daily contact with biomedical waste.

Segregation knowledge and practices

Overall, 257 participants (72.1%; 95% CI: 67.4%-76.4%) demonstrated correct knowledge of BMW segregation protocols, as assessed using a structured questionnaire aligned with the Biomedical Waste Management Rules, 2016 with correct responses most prevalent among nurses (n = 142, 76.3%) and least among doctors (n = 53, 63.0%). Each participant was presented with 20 waste items and asked to identify the appropriate color-coded disposal bin. A score of ≥75% correct responses (i.e., at least 15 out of 20 items) was considered indicative of correct knowledge.

The highest accuracy was observed for non-hazardous waste (Group I/II), while the most frequent errors occurred in identifying appropriate disposal methods for pharmaceutical and cytotoxic waste (Group IV).

Observation data revealed that correct segregation was followed in 84% of wards, but only 63% in outpatient departments. Color-coded bins were consistently present, but their use varied by department.

Risk perception scores

Risk perception was evaluated using a 5-point Likert scale (1 = No risk, 5 = Very high risk). Mean scores by perceived risk group (Table 2).

**Table 2:** Risk Perception Scores

Risk Group	Doctors	Nurses	Housekeepers	Overall Mean
Visitors	2.84	2.78	2.69	2.81
Patients	3.19	3.28	3.02	3.17
Own Health	3.97	4.10	3.80	3.95
Waste Workers	4.35	4.15	3.87	4.08
Environment	4.38	4.29	4.20	4.30

Doctors and nurses showed significantly higher risk perception than housekeeping staff (p < 0.05), especially regarding sharps-related risks and environmental contamination.

Needlestick and sharps injuries

A total of 41 needlestick or sharps-related injuries were reported in the past 12 months, with 20 cases (48.8%) among doctors, 16 cases (39.0%) among nurses, and five cases (12.2%) among housekeepers.

Of these, six injuries (14.6%) were directly linked to BMW handling. The majority occurred during patient care, particularly during venipuncture and intravenous line placement. Reporting compliance was higher among nurses at 151 (81.3%) compared to doctors at 51 (60.0%).

Training participation

Training attendance varied significantly across staff categories, with 76 (88.3%) housekeeping staff, 138 (74.2%) nurses, and 26 (31.0%) doctors having attended training.

Staff who attended training had significantly higher segregation knowledge and risk perception scores (p < 0.01). Housekeepers reported learning mainly through institutional orientation sessions, while doctors cited lack of time and perceived irrelevance as reasons for non-participation (Tables 3, 4).

**Table 3:** Biomedical Waste (BMW) Segregation Knowledge by Staff Category

Waste Type	Doctors (n=84)	Nurses (n=186)	Housekeepers (n=86)
Non-hazardous (Group I/II)	78.6%	81.7%	80.2%
Infectious (Group III)	63.1%	70.9%	68.6%
Hazardous (Group IV)	34.5%	57.5%	48.8%

**Table 4:** Risk Perception by Waste Category

Waste Group	Health Risk (Mean ± SD)	Environmental Risk (Mean ± SD)
Group I/II (Non-hazardous)	1.91 ± 0.58	2.62 ± 0.63
Group III (Infectious)	3.99 ± 0.70	4.05 ± 0.64
Group IV (Hazardous)	4.01 ± 0.68	4.14 ± 0.59

Association between staff category and key study variables

To examine relationships between staff categories and key variables, chi-square tests and one-way ANOVA were conducted. The association between staff designation and knowledge of BMW segregation was not statistically significant (χ² = 5.08, df = 2, p = 0.079), indicating that while nurses and housekeepers performed better than doctors, the differences were not large enough to reach statistical significance.

In contrast, training attendance varied significantly by role, with doctors showing much lower participation compared to nurses and housekeeping staff. This difference was highly statistically significant (χ² = 71.93, df = 2, p < 0.001). Similarly, the distribution of needlestick and sharps-related injuries also showed a statistically significant association with staff category (χ² = 16.75, df = 2, p < 0.001), with doctors reporting the highest number of incidents.

A one-way ANOVA was performed to compare risk perception scores across the three groups. The analysis found no statistically significant differences in overall perceived risk (F = 0.31, df = 2,9, p = 0.743), suggesting that despite differing roles, healthcare workers shared similar risk perceptions.

These findings underscore the need for uniform and targeted interventions, especially in training participation and injury prevention, to ensure consistent safety standards across all staff categories (Table 5).

**Table 5:** Statistical Analysis of Key Variables by Staff Category BMW: biomedical waste

Variable	Test Used	Test Statistic	Degrees of Freedom	p Value	Interpretation
BMW Segregation Knowledge	Chi-square	χ² = 5.08	df = 2	0.079	Not statistically significant
Training Attendance	Chi-square	χ² = 71.93	df = 2	< 0.001	Highly significant association
Sharps Injury Incidence	Chi-square	χ² = 16.75	df = 2	< 0.001	Statistically significant association
Risk Perception (overall)	One-way ANOVA	F = 0.31	df = 2, 9	0.743	No significant difference across categories

## Discussion

This study examined BMW management practices, occupational exposure, and risk perception among HCWs across four tertiary care teaching hospitals in South India. Using stratified sampling, we compared doctors, nurses, and housekeeping staff, revealing disparities in knowledge, compliance, injury reporting, and perception - gaps that mirror national and global challenges in biomedical waste handling.

Segregation knowledge and operational practice

A key focus of our study was to evaluate HCWs’ knowledge of BMW segregation per the Biomedical Waste Management Rules (2016) and assess how well this translated into real-world practices. While 72.1% of participants demonstrated correct knowledge, stratified analysis revealed that nurses scored highest (76.3%), followed by housekeepers (70.9%) and doctors (63%). These findings align with earlier studies in India by Patil and Shekdar [2] and Ferreira and Teixeira [7] abroad, where nursing and support staff - being more directly involved in daily waste handling - showed stronger operational knowledge.

Nevertheless, observational data highlighted gaps between knowledge and behavior, especially in high-pressure zones like emergency departments. Despite the presence of color-coded bins, inconsistent segregation and cross-contamination were frequent. Similar trends have been observed in Brazil and Libya, where logistical and behavioral factors such as time constraints, lack of bin availability, and inadequate supervision compromise waste compliance [3,8].

Misclassification was particularly high for Group IV waste (pharmaceutical and cytotoxic residues), often due to poor label visibility and staff confusion. The WHO's guidance underscores the need for continuous reinforcement of segregation through clear visual cues, frequent audits, and standard operating procedures (SOPs) tailored to department-specific waste types [9,10].

Risk perception and its determinants

Risk perception shapes compliance behavior. In our study, mean scores were highest for environmental impact (4.30) and worker exposure (4.08), with lower concern expressed for patient (3.17) and visitor risks (2.81). This reflects a compartmentalized understanding of biomedical risk-focused on direct rather than indirect exposure-echoing findings from both the Portugal study and other low-resource settings [7,11].

Housekeepers, though most exposed, perceived the least risk. This “perception paradox” is well documented, where vulnerable groups normalize occupational risk due to low health literacy or systemic neglect [12,13]. Standardized training alone may not resolve this; communication must be role-appropriate, multilingual, and reinforced with supportive workplace policies and incentives [4,14].

The US EPA and WHO emphasize that risk is not merely biological, it is also systemic [1,15]. Poor ventilation, inadequate temporary storage, and infrequent collection amplify indirect risks to all hospital stakeholders. These structural deficits were noted during our field inspections.

Sharps injuries and the challenge of underreporting

Our study recorded 41 needlestick or sharps injuries within a 12-month period. Doctors accounted for 20 (48.8%) of these incidents. Though most injuries occurred during direct patient care, six (14.6%) were linked to improper waste disposal, a trend that supports findings from the U.S. and UK, where sharps disposal errors contribute significantly to occupational injury rates [5,6,16].

Notably, only 51 (60%) doctors reported their most recent injury, compared to over 149 (80%) nurses. Underreporting compromises not only infection surveillance but also delays access to PEP [17,18]. The UNAIDS Global Report [19] and WHO’s exposure management protocols advocate universal access to incident reporting systems and timely follow-up.

We adopted the UK Environmental Agency’s model (2002) to estimate infection risk from sharps injuries, adjusting it using Indian seroprevalence data and vaccination rates. Although India lacks a standardized exposure risk calculator, this model aligns with Indian guidance from NACO and ICMR [20,21].

Training gaps and professional divide

Training participation was highest among housekeepers at 76 (88.3%), followed by nurses at 138 (74.2%), and lowest among doctors at 26 (31%) in terms of percentage. This inverse correlation with responsibility is troubling. Doctors, as primary waste generators, often bypass BMW training due to scheduling constraints or exemption from mandatory Continuing Medical Education (CME) modules [1,7].

Our data showed a strong positive correlation between training, segregation accuracy, and risk perception scores (p<0.05). These results reinforce findings from other studies which suggest that regular, context-specific training improves both awareness and adherence [14,22].

Trim and Elliott [23] also highlight that sharps safety training reduces injury incidence. Despite these benefits, several hospitals fail to make such modules compulsory for all levels of clinical staff - a gap that should be urgently addressed.

Environmental and institutional implications

Beyond occupational exposure, mismanaged BMW poses serious environmental risks. In India, missegregated waste is frequently incinerated, releasing hazardous pollutants like dioxins and furans, as documented in studies across India and the UK [1,2,24].

Our institutional observations revealed uncovered bins, improper signage, and poor ventilation in interim storage areas - conditions ripe for secondary contamination. Several departments also lacked waste collection logs or personal protective equipment (PPE) audits, indicating a compliance gap.

Technological solutions such as digital tracking of waste bins, barcode-linked segregation logs, and quarterly internal audits can drastically improve system transparency [25-27].

In parallel, hospitals can explore non-burn technologies like autoclaving, microwave sterilization, and nanophotocatalyst systems, which are safer and increasingly cost-effective in low and middle income countries (LMICs) [24,26,28].

Strengths and limitations

A key strength of our study was its multi-method design, integrating a validated questionnaire, field observations, and key informant interviews. The sample size (n=356) was statistically sufficient for subgroup analysis and covered diverse staff categories across four hospitals.

However, limitations include self-reported responses, which are susceptible to social desirability and recall bias; limited microbiological follow-up, restricting the estimation of actual seroconversion after sharps injuries; and although the study was multi-center, it was urban-focused, so the findings may not extrapolate to rural or under-resourced settings.

Nonetheless, the study offers a replicable model for institutional BMW audits and highlights policy and training gaps that remain under-addressed in many Indian healthcare settings.

Future recommendations

Recommendations include mandatory annual training for all healthcare staff including doctors with role-specific content. Regular internal audits and anonymous reporting systems should be implemented to capture and respond to noncompliance. Visual cues, multilingual signage and hands-on demonstrations can improve awareness in nonclinical staff. Biomedical waste modules should be integrated into medical and nursing undergraduate curricula. Digital tracking and incident reporting tools should be adopted, especially in high-risk areas like operating theatres and intensive care units. Routine review of standard operating procedures and procurement of personal protective equipment and color-coded bins will help support correct practices.

## Conclusions

This study highlights significant gaps in biomedical waste management knowledge, risk perception, and training among healthcare workers in a tertiary care teaching hospital in South India. While nurses and housekeeping staff showed better segregation practices, doctors had lower participation in training and awareness despite their central role in generating clinical waste. The gap between knowledge and practice was most evident in high-pressure areas like emergency and outpatient departments, pointing to the need for stronger system-level support. Although risk perception was high for environmental impact and waste worker safety, indirect risks to patients and visitors were often underestimated, especially by housekeeping staff. Needlestick injuries remain a common occupational hazard and underreporting adds to the safety challenges.

The findings call for urgent action through regular role-specific training for all staff, consistent internal audits, user-friendly reporting systems, and the integration of digital waste tracking tools. These interventions can improve compliance, protect healthcare workers from avoidable risks, and promote safer and more sustainable biomedical waste disposal practices across the Indian healthcare system.

## Appendices

Questionnaire: Assessment of Biomedical Waste Management and Risk Perception

Institution: Sree Balaji Medical College and Hospital Participant Code: ________ Date: ____ / ____ / ______

Title: Assessment of Biomedical Waste Management Practices and Risk Perception Among Healthcare Workers

Instructions to Participants:

Please answer all the questions honestly. Your responses will remain confidential and used only for academic and research purposes.

Domain 1: Demographic and Professional Information (10 Questions)

1. Age: ________

2. Gender: ☐ Male ☐ Female ☐ Other

3. Designation: ☐ Doctor ☐ Nurse ☐ Housekeeping staff

4. Department: _______________________

5. Years of service in this hospital: ______ years

6. Have you worked in other healthcare settings before? ☐ Yes ☐ No

7. Do you have daily contact with biomedical waste? ☐ Yes ☐ No

8. Have you had any formal training in biomedical waste management? ☐ Yes ☐ No

9. If yes, when was your last training session? ☐ <6 months ☐ 6-12 months ☐ >1 year

10. Have you read the BMW Management Rules 2016? ☐ Yes ☐ No

Domain 2: Knowledge of BMW Segregation (20 Questions)

Select the correct color-coded bin for each type of waste (one answer per question):

11. Soiled dressings and gauze

12. Used gloves

13. Plastic IV fluid bottles

14. Needles and blades

15. Human anatomical waste

16. Cytotoxic drugs

17. Expired medications

18. Microbiology lab cultures

19. Discarded blood bags

20. Glassware waste

21. General kitchen waste

22. Sharps with bodily fluid contamination

23.Syringes with fixed needles

24. Metallic implants

25. Empty vaccine vials

26. Chemical waste from cleaning

27. Contaminated cotton swabs

28. PPE kits used during patient care

29. Linen with blood stains

30. Saline bottle caps

Answer options per question:

☐ Yellow

☐ Red

☐ White (translucent)

☐ Blue

☐ Black (General)

☐ Not Sure

Domain 3: Risk Perception (15 Questions)

How risky do you believe each of the following are? Rate from 1 to 5 (1 = No risk, 5 = Very high risk)

31. Improper segregation of waste

32. Handling sharps without PPE

33. Exposure to airborne waste pathogens

34. Overflowing bins in patient areas

35. Disposal of cytotoxic waste into red bags

36. Contact with waste by visitors

37. Discarding contaminated plastics with general waste

38. Risk to environment from incineration

39. Risk of sharps injury while collecting waste

40. Risk of transmitting infection to patients

41. Risk of transmitting infection to yourself

42. Waste left in open corridors

43. Cross-contamination via reusable bins

44. Transport of waste through patient areas

45. Delays in waste pickup during holidays

Domain 4: Practices and Injury Reporting (10 Questions)

Answer Yes/No or select the appropriate option.

46. Have you ever experienced a needlestick or sharps injury? ☐ Yes ☐ No

47. If yes, how many times in the last 12 months? ☐ Once ☐ 2-3 times ☐ More than 3

48. Was the injury during BMW handling? ☐ Yes ☐ No

49. Did you report the injury? ☐ Yes ☐ No

50. Were you offered post-exposure prophylaxis (PEP)? ☐ Yes ☐ No

51. Do you consistently use PPE while handling BMW? ☐ Yes ☐ No

52. Are color-coded bins always available in your unit? ☐ Yes ☐ No

53. Have you ever seen coworkers improperly disposing BMW? ☐ Yes ☐ No

54. Does your unit conduct regular audits or BMW checks? ☐ Yes ☐ No

55. Would you like to receive additional BMW training? ☐ Yes ☐ No

## References

[1] (2014). Safe Management of Wastes From Health-Care Activities. 2nd ed. https://iris.who.int/bitstream/handle/10665/85349/9789241548564_eng.pdf?sequence=1.

[2] Patil AD, Shekdar AV (2001). Health-care waste management in India. J Environ Manage.

[3] Da Silva CE, Hoppe AE, Ravanello MM, Mello N (2005). Medical wastes management in the south of Brazil. Waste Manag.

[4] Sharma S (2010). Awareness about bio-medical waste management among healthcare personnel of some important medical centers in Agra. Int J Environ Sci Dev.

[5] Leigh JP, Wiatrowski WJ, Gillen M, Steenland NK (2008). Characteristics of persons and jobs with needlestick injuries in a national data set. Am J Infect Control.

[6] Gillen M, McNary J, Lewis J (2003). Sharps-related injuries in California healthcare facilities: pilot study results from the Sharps Injury Surveillance Registry. Infect Control Hosp Epidemiol.

[7] Ferreira V, Teixeira MR (2010). Healthcare waste management practices and risk perceptions: findings from hospitals in the Algarve region, Portugal. Waste Manag.

[8] Sawalem M, Selic E, Herbell JD (2009). Hospital waste management in Libya: a case study. Waste Manag.

[9] Easty AC, Coakley N, Cheng R, Cividino M, Savage P, Tozer R, White RE (2015). Safe handling of cytotoxics: guideline recommendations. Curr Oncol.

[10] Prüss A, Giroult E, Rushbrook P (1999). Safe Management of Wastes From Health-Care Activities. https://apps.who.int/iris/handle/10665/42175.

[11] Manzoor J, Sharma M (2019). Impact of biomedical waste on environment and human health. Environ Claims J.

[12] Ali M, Wang W, Chaudhry N, Geng Y (2017). Hospital waste management in developing countries: a mini review. Waste Manag Res.

[13] Khalid S, Haq N, Sabiha ZU, Latif A, Khan MA, Iqbal J, Yousaf N (2021). Current practices of waste management in teaching hospitals and presence of incinerators in densely populated areas. BMC Public Health.

[14] Akkajit P, Romin H, Assawadithalerd M (2020). Assessment of knowledge, attitude, and practice in respect of medical waste management among healthcare workers in clinics. J Environ Public Health.

[15] (1992). Guidelines for exposure assessment. DC: US EPA.

[16] Blenkharn JI (2006). Standards of clinical waste management in UK hospitals. J Hosp Infect.

[17] Beltrami EM, Williams IT, Shapiro CN, Chamberland ME (2000). Risk and management of blood-borne infections in health care workers. Clin Microbiol Rev.

[18] (2001). Updated guidelines for management of occupational exposures to HBV, HCV, and HIV. MMWR Recomm Rep.

[19] (2008). Report on the Global AIDS Epidemic. https://data.unaids.org/pub/globalreport/2008/jc1510_2008_global_report_pp29_62_en.pdf.

[20] Rapiti E, Prüss-Üstün A, Hutin Y, Campbell-Lendrum D, Corvalán C, Woodward A (2005). Sharps Injuries. Assessing the Burden of Disease From Sharps Injuries to Health-Care Workers at National and Local Levels. http://iris.who.int/bitstream/handle/10665/43051/924159232X.pdf.

[21] Elder A, Paterson C (2006). Sharps injuries in UK health care: a review of injury rates, viral transmission and potential efficacy of safety devices. Occup Med (Lond).

[22] Joshi SC, Diwan V, Tamhankar AJ (2015). Staff perception on biomedical or health care waste management: a qualitative study in a rural tertiary care hospital in India. PLoS One.

[23] Trim JC, Elliott TS (2003). A review of sharps injuries and preventative strategies. J Hosp Infect.

[24] Diaz LF, Savage GM, Eggerth LL (2005). Alternatives for the treatment and disposal of healthcare wastes in developing countries. Waste Manag.

[25] Singh H, Jaiswal A, Tiwari R, Gupta N (2024). Harnessing the foundation of biomedical waste management for fostering public health. Discov Appl Sci.

[26] Hooshmand S, Kargozar S, Ghorbani A, Darroudi M, Keshavarz M, Baino F, Kim HW (2020). Biomedical waste management by using nanophotocatalysts: the need for new options. Materials (Basel).

[27] Patil PM, Bohara RA (2020). Nanoparticles impact in biomedical waste management. Waste Manag Res.

[28] Thakur V, Ramesh A (2017). Healthcare waste disposal strategy selection using grey-AHP approach. Benchmarking.

